# Harmonization of standard uptake values across different positron emission tomography/computed tomography systems and different reconstruction algorithms: validation in oncology patients

**DOI:** 10.1186/s40658-023-00540-z

**Published:** 2023-03-15

**Authors:** Yufei Song, Xiangxi Meng, Zhen Cao, Wei Zhao, Yan Zhang, Rui Guo, Xin Zhou, Zhi Yang, Nan Li

**Affiliations:** 1 Key Laboratory of Carcinogenesis and Translational Research (Ministry of Education/Beijing), Key Laboratory for Research and Evaluation of Radiopharmaceuticals (National Medical Products Administration), Department of Nuclear Medicine, Peking University Cancer Hospital and Institute, Beijing, China; 2 Siemens Healthineers Ltd., Shanghai, China

**Keywords:** PET/CT, EQ.PET, SUV harmonization

## Abstract

**Background:**

EQ.PET is a software package that overcomes the reconstruction-dependent variation of standard uptake values (SUV). In this study, we validated the use of EQ.PET for harmonizing SUVs between different positron emission tomography/computed tomography (PET/CT) systems and reconstruction algorithms.

**Methods:**

In this retrospective study, 49 patients with various cancers were scanned on a Biograph mCT (mCT) or Gemini TF 16 (Gemini) after [^18^F]FDG injections. Three groups of patient data were collected: Group 1, patients scanned on mCT or Gemini with data reconstructed using two parameters; Group 2, patients scanned twice on different PET scanners (interval between two scans, 68.9 ± 41.4 days); and Group 3, patients scanned twice using mCT with data reconstructed using different algorithms (interval between two scans, 109.5 ± 60.6 days). The SUVs of the lesions and background were measured, and the tumor-to-background ratios (TBRs) were calculated. In addition, the consistency between the two reconstruction algorithms and confounding factors were evaluated.

**Results:**

In Group 1, the consistency of SUV and TBR between different reconstruction algorithms improved when the EQ.PET filter was applied. In Group 2, by comparing ΔSUV, ΔSUV%, ΔTBR, and ΔTBR% with and without the EQ.PET, the results showed significant differences (*P* < 0.05). In Group 3, Bland–Altman analysis of ΔSUV with EQ.PET showed an improved consistency relative to that without EQ.PET.

**Conclusions:**

EQ.PET is an efficient tool to harmonize SUVs and TBRs across different reconstruction algorithms. Patients could benefit from the harmonized SUV, ΔSUV, and ΔSUV% for therapy responses and follow-up evaluations.

**Supplementary Information:**

The online version contains supplementary material available at 10.1186/s40658-023-00540-z.

## Background

[^18^F]Fluorodeoxyglucose ([^18^F]FDG) positron emission tomography (PET) is a well-known tool for tumor diagnosis, staging, and therapy monitoring [1]. The standard uptake value (SUV) is the most widely used quantitative parameter of PET imaging [2]. Because of the impact from biological, physical, and technical factors [3], the reproducibility of SUVs remains problematic. In addition, the reconstruction algorithms in PET can vary, leading to large differences in SUVs. The glucose metabolic rate acquired from dynamic [^18^F]FDG-PET scans is often considered to be the gold standard of quantitative PET imaging [4] but is not commonly used in clinics. Despite its limitations, SUV is widely used in clinical practice due to its straightforwardness.

A standard solution for SUV comparison in tumor PET imaging provided by the European Association of Nuclear Medicine (EANM) is harmonizing patient preparation, PET acquisition, and image reconstruction algorithms [5, 6]. For cross-system comparisons with reconstruction-dependent variations, an additional filtering step of the reconstructed images is recommended to achieve harmonized SUVs [7]. EQ.PET is a software solution that applies a spatial filter to each set of acquisition and reconstruction parameters, based on the procedure recommended by EANM, therefore it does not rely on multiple reconstructions [8].A series of studies demonstrated both the EANM-recommended solution and EQ.PET could generate harmonized SUVs that overcome the reconstruction-dependent variations in multicenter quantitative PET studies and in therapy monitoring with different reconstruction algorithms [8–14].

Aiming at validating the performance of EQ.PET on the SUV harmonization of different scanners and reconstruction algorithms, a retrospective study was conducted involving PET/CT systems from two vendors: Gemini TF 16 (Philips Medical Systems) and Biograph mCT (Siemens Healthineers, Knoxville, USA). SUVs acquired from these two systems are not directly comparable, making it difficult to establish uniform standards for diagnosis, staging, or therapy monitoring. Patients may be scanned on different systems because of necessary system upgrades or maintenance. In addition, patients receiving follow-up scans in different centers also make assessment difficult. In this study of oncology patients, we investigated the feasibility of harmonizing SUVs from different reconstruction algorithms from two different PET/CT systems using the EQ.PET software.

## Materials and methods

### PET/CT scanners

Phantom and patient imaging data were collected on two PET/CT scanners: Biograph mCT (mCT) (Siemens Healthineers, Knoxville, USA) and Gemini TF 16 (Gemini) (Philips Medical Systems). The daily quality controls of each PET/CT scanner were performed using a ^68^Ge calibration source. In addition, the quarterly cross-calibration was performed according to the EANM guidelines [5], and all clocks were synchronized weekly.

### Phantom preparation

A NEMA/IEC torso phantom with six coplanar spheres with internal diameters of 10, 13, 17, 22, 28, and 37 mm was used. All spheres were filled with [^18^F]FDG solution at an initial radioactivity concentration of 38.9 kBq/mL, and the ratio of radioactivity concentration between the sphere and the background was 10:1.

### Phantom analysis for EQ.PET filter calculation

As described in a previously published study, phantom analyses were performed to acquire the optimal EQ.PET filters for each scanner and reconstruction algorithm [15]. Briefly, volumes of interest (VOIs) corresponding to the dimensions of each sphere were drawn, and the voxels with the maximum activity (kBq/ml) were determined. Next, the ratio of measured-to-true activity concentration for each sphere was calculated as recovery coefficients (RCs). The RCs applied with a range of EQ.PET filter values were compared to a set of reference RCs [6] to calculate the root mean square error (RMSE). The spatial filter that minimized the RMSE was then selected as the optimal EQ.PET filter for the specific scanner and reconstruction algorithm.

### Patient information

In this retrospective study, 49 patients with various cancer types were scanned at the Peking University Cancer Hospital from August 1, 2019, to December 30, 2020. The three groups of patients in this study were: **Group 1**, 23 patients scanned on mCT, and fifteen patients scanned on Gemini with PET images reconstructed using two different algorithms; **Group 2**, eleven patients scanned twice on different scanners (interval between two scans, 68.9 ± 41.4 days) for follow-up or therapy monitoring; and **Group 3**, fourteen patients from **Group 1** scanned twice on mCT (interval between two scans, 109.5 ± 60.6 days) for follow-up or treatment monitoring. The patient demographics of each group are summarized in Table 1 and Additional file 1: Tables S1–S3.

**Table 1:** Patient demographics

Group No.	PET scanner	Gender (F/M*)	Age (y^#^)	Cancer type	Patients included	Lesions included
1	mCT	10/13	60.04 ± 8.64	Lung cancer	15	67
				Hepatic cancer	2	16
				Gallbladder carcinoma	1	18
				Esophageal cancer	1	4
				Pancreatic cancer	1	9
				Non-Hodgkin’s lymphoma	1	9
				Melanoma	1	2
				Rectal carcinoma	1	3
				Diffuse large B cell lymphoma	1	5
1	Gemini	5/10	60.33 ± 9.09	Lung cancer	5	19
				Non-Hodgkin’s lymphoma	4	31
				Esophageal cancer	2	8
				Colon cancer	2	11
				Diffuse large B cell lymphoma	1	6
				Breast cancer	1	4
2	mCT & Gemini	8/3	52.09 ± 14.79	Non-Hodgkin’s lymphoma	6	19
				Hodgkin’s lymphoma	2	2
				Diffuse large B cell lymphoma	1	3
				Lung cancer	1	1
				Esophageal carcinoma	1	2
3	mCT	7/7	59 ± 9.21	Lung cancer	11	19
				Non-Hodgkin’s lymphoma	1	9
				Rectal adenocarcinoma	1	3
				Melanoma	1	2

*F: female, M: male; ^#^y: years

### PET/CT acquisition and reconstruction algorithms

Both the phantom and patients were scanned using CT with subsequent PET scans with clinical scan parameters. The scan parameters and reconstruction algorithms for each system are summarized in Table 2. Patients fasted for over six hours prior to the injection with [^18^F]FDG (8.41 ± 1.05 mCi, IV) and were scanned from the skull base to the mid-thigh approximately one hour post-injection.

**Table 2:** PET/CT scan and reconstruction algorithms

PET Scanner		Biograph mCT		Gemini TF 16	
CT	Voltage/Intensity	120 kV/ ref mAs		120 kV/ 100 mAs	
	Collimation/pitch	16 × 1.2 mm/pitch 0.8		16 × 1.5 mm/pitch 0.813	
PET scan parameter	Bed motion speed	FlowMotion mode 2 mm/s (~ 100 s/bed)		40 s/bed	
PET reconstruction algorithm	Reconstruction algorithm	OSEM	OSEM + PSF + TOF (*Clinical*)	BLOB-OS-TOF (*Clinical*)	BLOB-OS-TOF
	Iterations/Subsets	2/24	2/21	3/33	3/33
	Smoothing	No	5 mm	Normal*	Smooth A*
	Matrix	200 × 200	200 × 200	144 × 144	144 × 144
	EQ.PET filter (mm)	8	7.8	4.9	4.4

*The ‘normal’ and ‘smooth A’ smoothing options correspond to a relaxation parameter of 1.0 and 0.6 in the BLOB-OS-TOF algorithm, respectively

PET images were reconstructed using different reconstruction algorithms. For the mCT PET scans, the clinical algorithm was ordered subset expectation maximization (OSEM) (2 iterations and 21 subsets) + PSF + TOF + 5 mm Gaussian filter (noted as *clinical*). In addition, the OSEM (two iterations and 24 subsets) (noted as *OSEM*) algorithm was also used for comparison if raw data were available. The matrix size was 200 × 200 for both reconstruction algorithms. For the Gemini PET scans, the algorithm used clinically was BLOB-OS-TOF (2 iterations and 33 subsets) with a ‘normal’ smoothing setting (noted as *clinical*). In contrast, BLOB-OS-TOF (3 iterations and 33 subsets) with a ‘smooth A’ smoothing setting (noted as *smooth A*) was used for comparison if raw data were available. The matrix size was 144 × 144 for both reconstruction algorithms. Scatter and attenuation corrections were applied in all reconstruction algorithms.

### PET/CT image analysis

All PET/CT image analyses were performed on a Syngo Via workstation (Siemens Medical Solutions) by the same reader (R.G.).

### Patient data analysis

Semi-quantitative analyses were performed on all patient data. For the CT images, the long axis of each lesion was measured and recorded as the lesion size. For the PET images, VOIs with a 50% isocontour were drawn on representative lesions (including primary and metastatic lesions), and VOIs with fixed diameters (*d*) were drawn on livers (*d* = 30 mm) and aortas (*d* = 10 mm). The EQ.PET filter was applied for SUV harmonization (SUV_eq_). In addition, SUVs with and without harmonization were automatically displayed. The tumor-to-background ratios (TBR), including the tumor-to-liver ratio (T/L), and tumor-to-blood ratio (T/B) were calculated and recorded. Lastly, for patients scanned twice, ΔSUV_max_, ΔSUV_max%_, ΔTBR, and ΔTBR% were calculated as follows:

$$\begin{aligned} \Delta {\text{SUV}}_{\max } & = {\text{SUV}}_{\max } \left( {2{\text{nd}}\;{\text{PET}}\;{\text{scan}}} \right) - {\text{SUV}}_{\max } \left( {1{\text{st}}\;{\text{PET}}\;{\text{scan}}} \right) \\ \Delta {\text{SUV}}_{\max } \% & = \Delta {\text{SUV}}_{\max } /{\text{SUV}}_{\max } \left( {1{\text{st}}\;{\text{PET}}\;{\text{scan}}} \right) \times 100\% \\ {\text{TBR}} & = {\text{SUV}}_{\max } \left( {{\text{lesion}}} \right)/{\text{SUV}}_{{{\text{mean}}}} ({\text{background}}\;{\text{VOI}}) \\ \Delta {\text{TBR}} & = {\text{TBR}}\left( {2{\text{nd}}\;{\text{PET}}\;{\text{scan}}} \right) - {\text{TBR}}\left( {1{\text{st}}\;{\text{PET}}\;{\text{scan}}} \right) \\ \Delta {\text{TBR}}\% & = \Delta {\text{TBR}}/{\text{TBR}}\left( {1{\text{st}}\;{\text{PET}}\;{\text{scan}}} \right) \times 100\% \\ \end{aligned}$$

### Statistical analysis

For EQ.PET filter selection, the RCs of different reconstruction algorithms were compared with EANM-expected RCs [6] by calculating the RMSE. Quantitative data from the patients were presented as means ± SD. Bland–Altman plot analyses were performed to evaluate consistency. The ratios of the SUVs of two different reconstruction algorithms were compared using Student’s *t*-test. *P* values less than 0.05 were considered statistically significant. Lastly, graphs were analyzed using GraphPad Prism 7.0.

## Results

### Phantom analysis

Phantom data were analyzed for EQ.PET filter selection (Additional file 1: Fig. S1). The optimal EQ.PET filter sizes for the clinical and OSEM reconstruction algorithms of mCT were 7.8 mm and 8.0 mm, respectively. In addition, the optimal EQ.PET filter sizes for Gemini’s *clinical* and *Smooth A* algorithms were 4.9 mm and 4.4 mm, respectively. The optimal EQ.PET filters were applied to patient data for each specific scanner and reconstruction algorithm.

### Validation of the EQ.PET software in patient data with different reconstruction algorithms

**Fig. 1:**
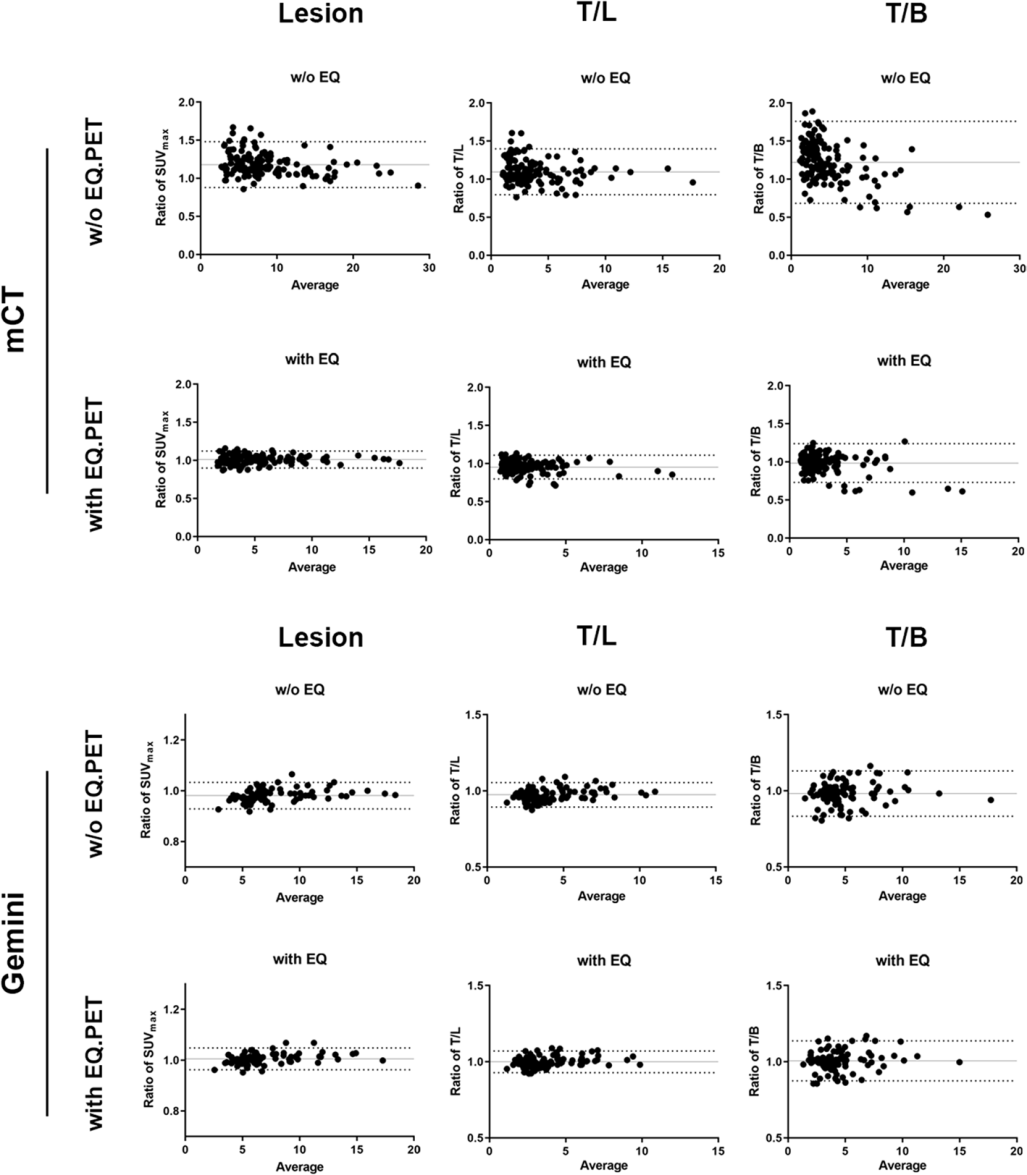
Bland–Altman analyses of SUV in lesions and TBR between data reconstructed using different reconstruction algorithms acquired on mCT (A) and Gemini (B) in **Group 1**

Patient data from **Group 1** were reconstructed using different reconstruction algorithms to validate that the EQ.PET software could overcome the reconstruction-dependent variability from mCT and Gemini, respectively. The Bland–Altman analysis was performed to evaluate the consistency of SUVs in the lesions and TBR with or without the EQ.PET filter (Fig. 1). As summarized in Table 3, for data acquired on mCT, the mean ratio between the clinical and OSEM reconstruction algorithms, and the 95% limits of agreement (95%LoA) in the lesions, T/L, and T/B without the EQ.PET filter were 1.18 (95% LoA: 0.88–1.48), 1.10 (95% LoA: 0.80–1.40), and 1.22 (95% LoA: 0.68–1.76), respectively. With the EQ.PET filter, these values were reduced to 1.01 (95% LoA: 0.90–1.12), 0.95 (95% LoA: 0.80–1.11), and 0.98 (95% LoA: 0.73–1.24), respectively. Patients scanned on Gemini with data reconstructed using the *clinical* and *Smooth A* were also analyzed. The results of the Bland–Altman analysis were similar.

**Table 3:** Ratios of measured values in Group 1 image pairs

Mean (95% LoA)	Lesion SUVmax	T/L	T/B
*mCT*			
w/o EQ	1.18 (0.88–1.48)	1.10 (0.80–1.40)	1.22 (0.68–1.76)
with EQ	1.01 (0.90–1.12)	0.95 (0.80–1.11)	0.98 (0.73–1.24)
*Gemini*			
w/o EQ	0.98 (0.93–1.03)	0.97 (0.89–1.06)	0.98 (0.83–1.13)
with EQ	1.01 (0.96–1.05)	1.00 (0.93–1.07)	1.01 (0.87–1.14)

95% LoA: limits of agreement

**Fig. 2:**
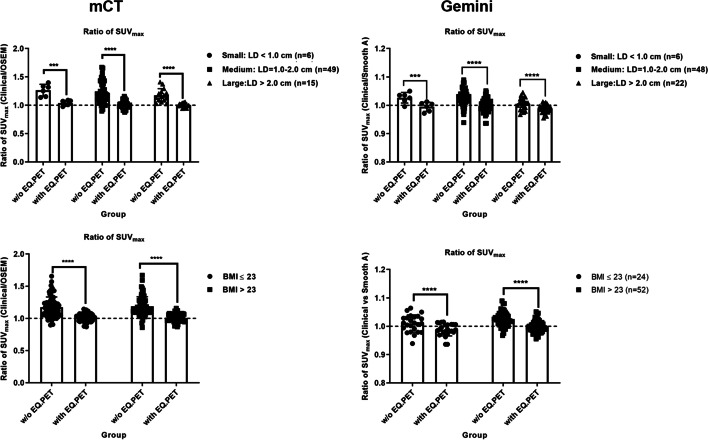
Impact of lesion size (upper panel) and BMI (lower panel) on SUVs acquired on mCT and Gemini

To further evaluate the confounding factors, the ratio of SUV_max_ in lesions between two reconstruction algorithms was separated based on lesion sizes and patient BMI levels (the obesity standard for Asian adults was applied [16]). As shown in Fig. 2 and Table 4, the ratio of SUV_max_ between the two reconstruction algorithms was significantly reduced after the application of the EQ.PET filter in each subgroup in mCT and Gemini.

**Table 4:** Ratios of SUVmax for image pairs of Group 1 (grouped by lesion size and patient BMI)

Mean ± SD	Lesion size			BMI	
	Small	Medium	Large	< 23	> 23
*mCT*					
w/o EQ	1.27 ± 0.10	1.22 ± 0.18	1.18 ± 0.12	1.17 ± 0.16	1.19 ± 0.15
with EQ	1.04 ± 0.05	1.02 ± 0.06	1.00 ± 0.04	1.01 ± 0.06	1.01 ± 0.06
*Gemini*					
w/o EQ	1.03 ± 0.02	1.03 ± 0.03	1.01 ± 0.02	1.01 ± 0.03	1.03 ± 0.02
with EQ	0.99 ± 0.01	0.99 ± 0.02	0.99 ± 0.002	0.99 ± 0.02	1.00 ± 0.02

### Validation of the EQ.PET software in patient data for therapy monitoring or follow-up

We analyzed patient data from **Group 2** and **Group 3** for therapy monitoring or follow-up. Patients in **Group 2** were scanned first on mCT and then on Gemini, or vice versa (interval between two scans: 68.9 ± 41.4 days). Patients in **Group 3** were scanned twice on mCT (interval between scans: 109.5 ± 60.6 days). The patient demographics are summarized in Table 2 and detailed in Additional file 1: Tables S2 and S3.

**Fig. 3:**
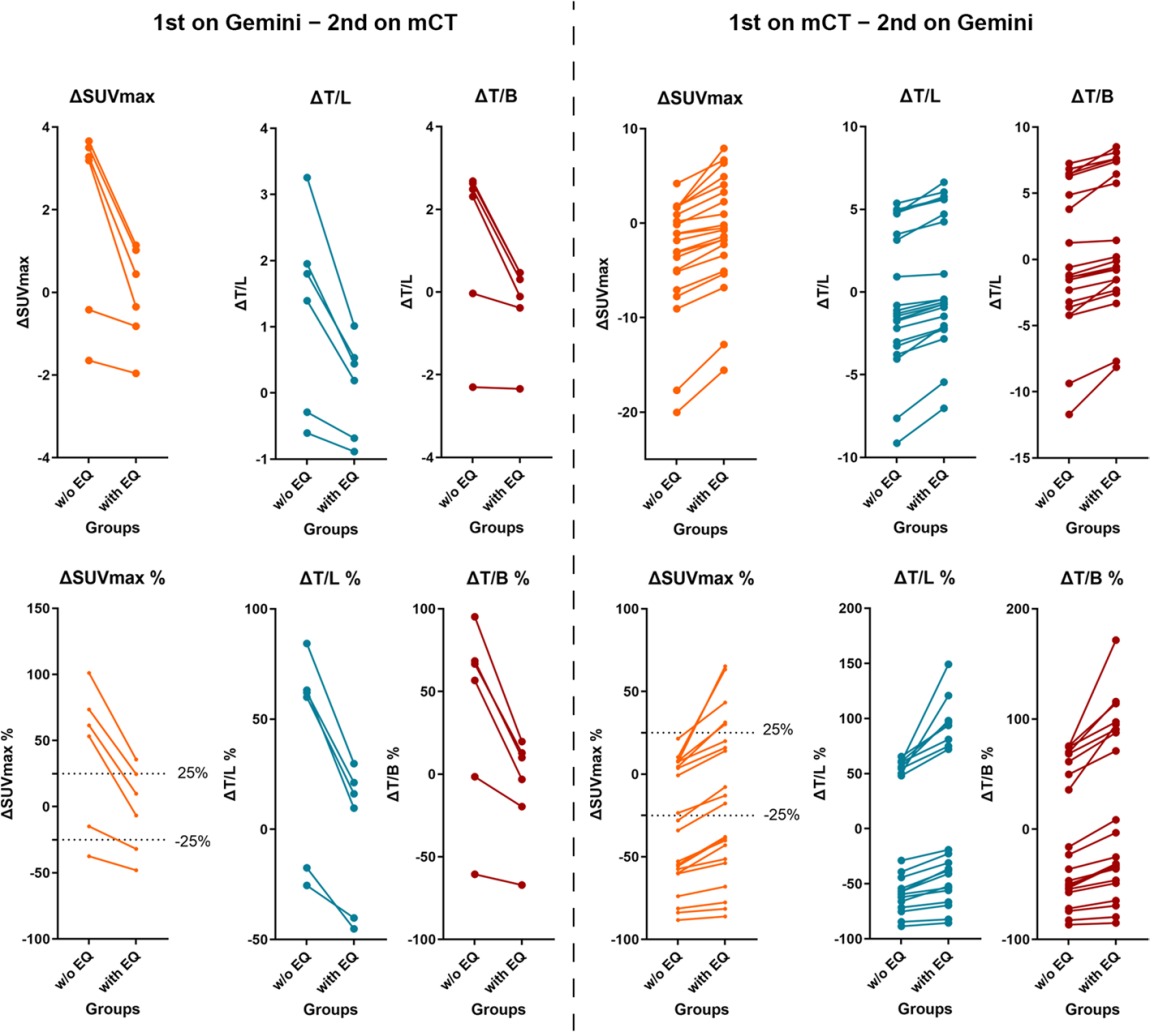
Comparison between ΔSUV, ΔSUV%, ΔTBR, and ΔTBR% in patients scanned twice; first on Gemini and subsequently on mCT (left panel), or first on mCT and then on Gemini (right panel) for follow-up or therapy monitoring in **Group 2**

**Fig. 4:**
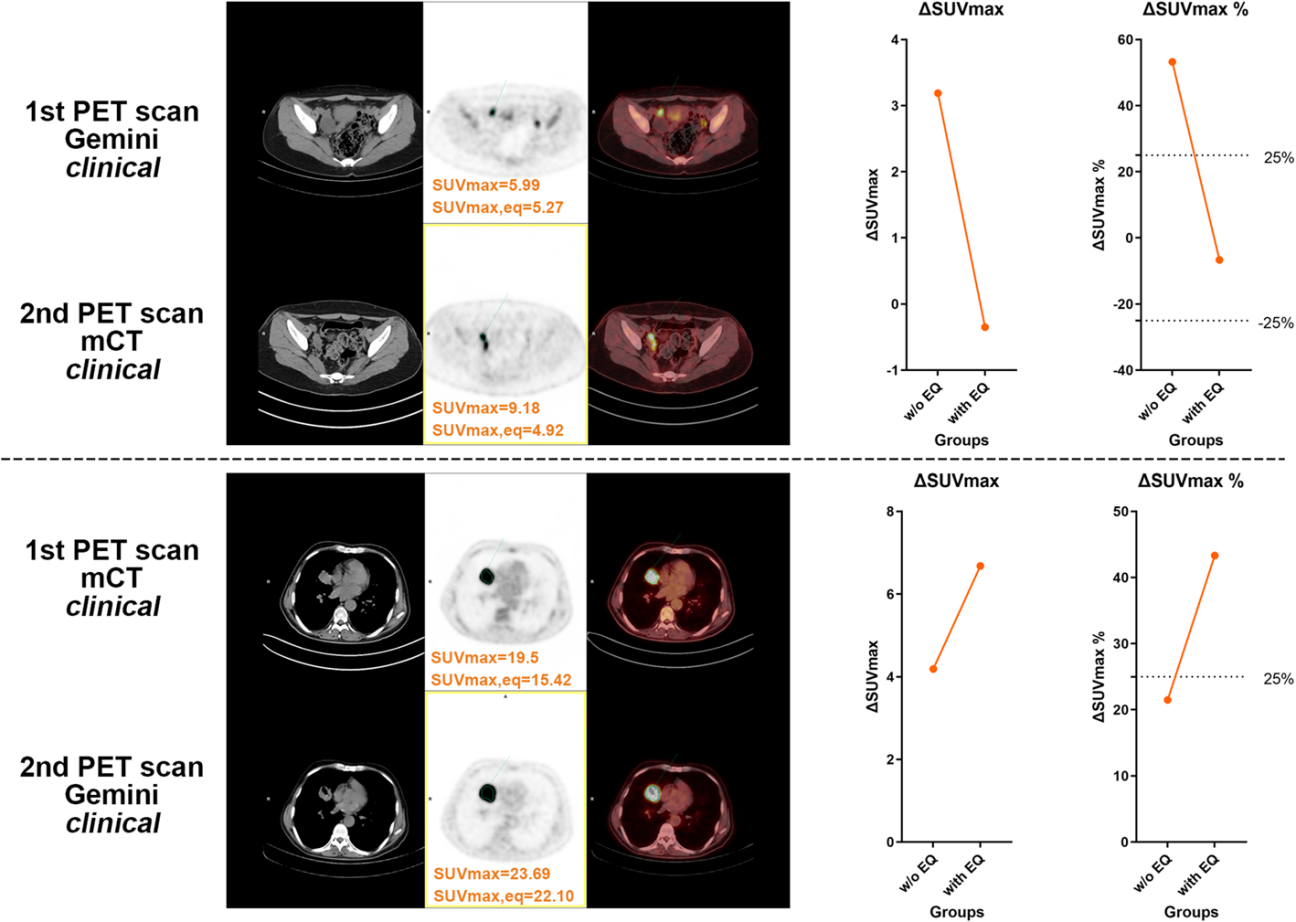
Representative PET/CT images and quantitative analyses of patients from Group 2. Patients were scanned first on Gemini and subsequently on mCT (upper panel), or first on mCT and then on Gemini (lower panel). All scanned data were reconstructed using the clinical algorithms of each PET system. ΔSUV_max_ and ΔSUV_max%_ were notably changed when applying the EQ.PET filter

Twenty-seven lesions were analyzed in **Group 2**. Data from both PET scans were reconstructed using clinical parameters. As shown in Fig. 3, ΔSUV, ΔSUV%, ΔTBR, and ΔTBR% showed significant differences between data with and without the EQ.PET filter. Figure 4 shows the patients’ representative PET/CT images in **Group 2**, demonstrating that the results of the therapy response change when the EQ.PET filter is applied.

**Fig. 5:**
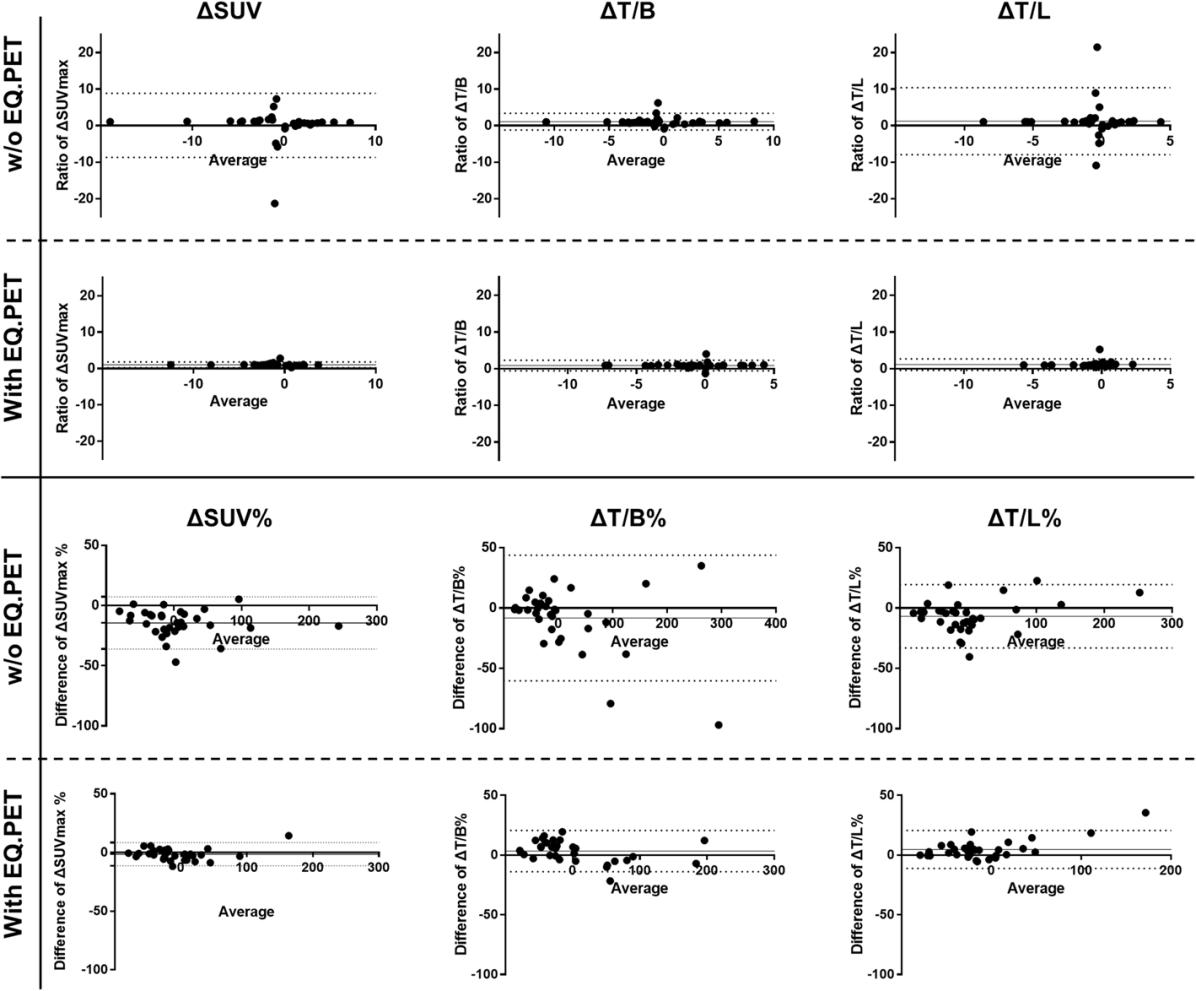
Bland–Altman analyses of ΔSUV, ΔSUV%, ΔTBR, and ΔTBR% between the two reconstruction algorithms (*clinical*-*clinical* vs. *OSEM*-*clinical*) in patients scanned twice on mCT in **Group 3** (the first PET scan was reconstructed using the clinical algorithm, and the subsequent PET scan was reconstructed using the *clinical* and *OSEM* algorithms.)

**Fig. 6:**
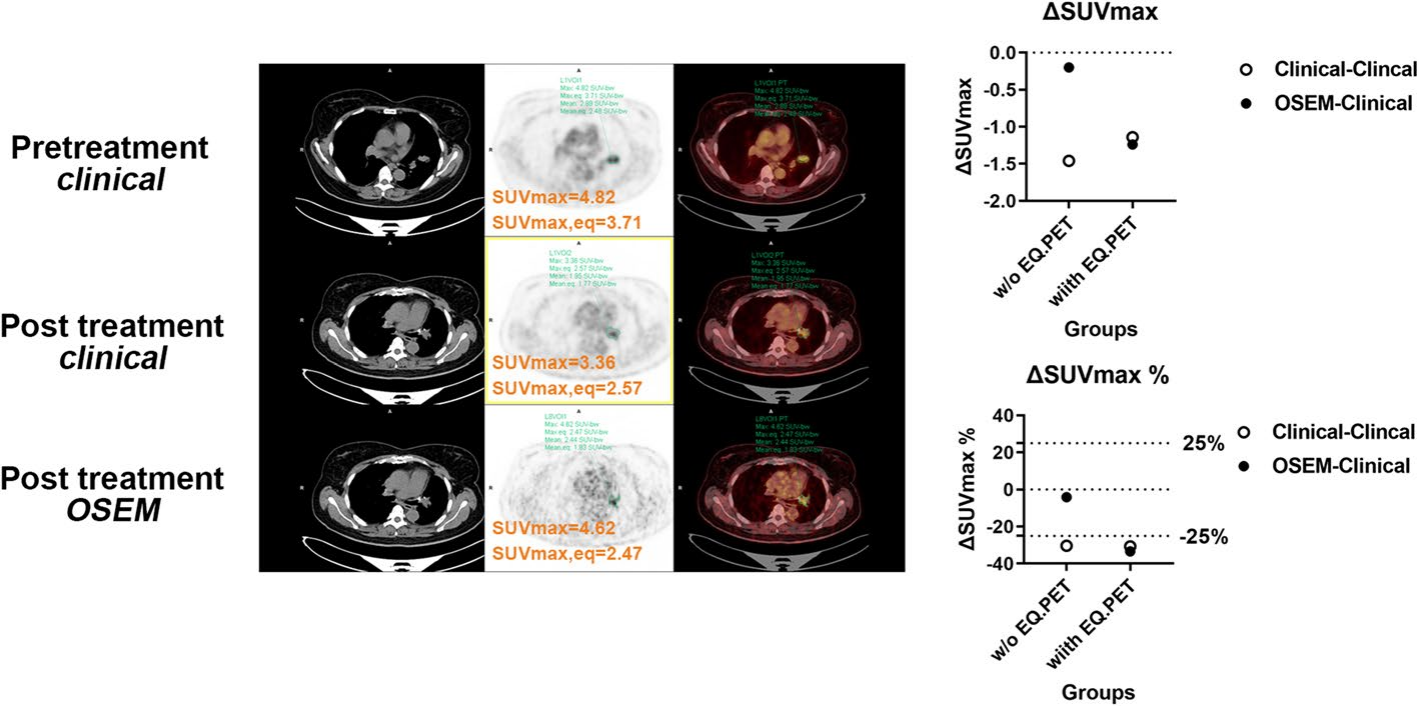
Representative PET/CT images and quantitative analyses of patients from **Group 3**. The patient was scanned before and after treatment. Pre-treatment scan data were reconstructed using the *clinical* reconstruction algorithm (upper panel). Post-treatment scan data were reconstructed using the *clinical* (middle panel) and *OSEM* (lower panel) algorithms. Without EQ.PET, the ΔSUV_max_ and ΔSUV_max_% of the two different reconstruction algorithms showed different therapy response results. With EQ.PET, the ΔSUV_max_ and ΔSUV_max%_ of the two different reconstruction algorithms showed consistent results

For **Group 3**, a total of 33 lesions were analyzed. The first PET scan data were reconstructed using the *clinical* algorithm, and the raw data from the second PET scan were reconstructed using both the *clinical* and the *OSEM* algorithms. Figure 5 shows the mean ratios of ΔSUV and ΔSUV% without EQ.PET were 0.07 (95% LoA: − 8.70–8.84) and − 14.38 (95% LoA: − 36.10–7.34). After the application of the EQ.PET filter, the mean ratios of ΔSUV and ΔSUV% changed to 1.02 (95% LoA: 0.23–1.81) and − 1.41 (95% LoA: − 11.27–8.46). Notably, the interval of 95% LoA was narrowed with EQ.PET. Figure 6 shows the representative PET/CT images of patients from **Group 3**. Lastly, the big differences between ΔSUV and ΔSUV% were reduced by the EQ/PET filter.

## Discussion

The comparability of SUVs is essential for PET imaging and the quantitative analysis. Many factors can impact the accuracy and reproducibility of SUVs. A harmonized SUV could be achieved by following EANM guidelines with unified patient preparation, PET acquisition, and reconstruction algorithms [5, 6]. However, in clinical practice, reconstruction algorithms of PET systems from different vendors vary. The reconstruction algorithm differs in various models and product lines, even for the same vendor. These differences make it difficult to overcome reconstruction-dependent variations across systems. With two PET scanners using different reconstruction algorithms in the center, we are forced to find solutions to harmonize SUVs across the two PET systems.

In **Group 1**, we used EQ.PET to harmonize the SUVs of different reconstruction algorithms from the same PET system. The raw data of each PET system were reconstructed using two different algorithms. TBR is a relative ratio of lesion uptake and background uptake, which could eliminate the influence of injection activity, and is recommended to be an alternative to SUV for malignancy identification and therapy monitoring [17–20]. To the best of our knowledge, the feasibility of EQ.PET in harmonization of TBR has not been evaluated in previous studies. Based on this, SUVs and TBRs with and without EQ.PET between different reconstruction algorithms were compared by performing Bland–Altman analysis. Our results (Fig. 1 and Table 3) showed that the differences of SUVs and TBRs between the two reconstruction algorithms from mCT can be reduced through the application of EQ.PET. Moreover, with minor differences between the two reconstruction algorithms, harmonization of SUVs seems to be dispensable for data from Gemini.

Considering that other factors may impact the feasibility of SUV harmonization using EQ.PET, the ratio of SUVs between the two reconstruction algorithms was compared by separating data into different lesion sizes and patient BMI sub-groups. As shown in Fig. 2 and Table 4, the ratios of SUVs in each subgroup were significantly reduced after applying the EQ.PET filter in mCT and Gemini. These results were consistent with previous studies [8] and demonstrated that the EQ.PET technology can be used to overcome reconstruction-dependent variations, regardless of lesion size or patient BMI. In addition, the differences in SUV between the two reconstruction algorithms from Gemini can be improved after SUV harmonization with respect to lesion size and patient BMI. These results convinced us to apply the EQ.PET filter in Gemini when comparing SUVs of different reconstruction algorithms.

As mentioned previously, SUV comparisons across two PET systems are a significant problem, particularly for therapy monitoring and follow-up examinations. Therefore, the feasibility of EQ.PET in therapy monitoring was investigated in two groups of patients (**Group 2** and **Group 3**). For metabolic therapy monitoring, percentage change of SUV (ΔSUV%) before and after treatment is the most used standard for therapy response classification (for example, the EORTC criteria set the reduction or increase of 25% in the sum SUV of lesions as the response evaluation standard). Changes in TBR (ΔTBR) or percentage changes in TBR (ΔTBR%) were reported to be more reliable in therapy response evaluation [21]. As far as we know, the feasibility of EQ.PET in harmonization ΔSUV, ΔSUV%, ΔTBR, and ΔTBR% has not been reported, although ΔSUL and ΔSUL% has been evaluated in some previous studies [11, 12, 22].

In **Group 2**, patients scanned on different PET scanners for follow-up examinations or therapy monitoring were analyzed. As shown in Figs. 3 and 4, significant differences were observed in all measured quantitative parameters (ΔSUV, ΔSUV%, ΔT/L, ΔT/L%, ΔT/B, and ΔT/B%) between data with and without the EQ.PET filter. These findings indicated that the follow-up or therapy monitoring results can be changed by harmonizing SUVs, possibly impacting patient management.

We evaluated patients scanned twice using mCT in **Group 3**. Figure 5 and Additional file 1: Table S4 show that the differences of ΔSUV, ΔSUV%, ΔTBR, and ΔTBR% between the same reconstruction (*clinical*-*clinical*) and different reconstructions (*OSEM*-*clinical*) could be reduced by applying the EQ.PET filter, demonstrating the metabolic therapy evaluation results were comparable between different reconstructions after harmonization. Furthermore, significant differences were not observed in ΔSUV, ΔSUV%, ΔTBR, and ΔTBR% of the same reconstruction (*clinical*-*clinical*) between data with and without harmonization (Additional file 1: Fig. S2). This may convince us that the comparable ΔSUV, ΔSUV%, ΔTBR, and ΔTBR% between the same reconstruction and different reconstructions after harmonization could lead to a consistent therapy response classification. Therefore, SUV harmonization using EQ.PET is an effective solution to reach consistent therapy response results between different PET systems and different reconstructions. The benefits of harmonizing the SUV in the therapy response evaluation have been investigated with various criteria, such as EORTC, PERCIST, and the Deauville score [10–13, 23]. Although our therapy response results were not classified according to specific criteria, our findings were consistent with these studies.

SUV_peak_ is shown to be a robust metric in the quantification of PET scans [24, 25], and EQ,PET is also capable of harmonization using SUV_peak_. We performed a Bland–Altman analysis using SUV_peak_ on **Group 1**. As shown in Additional file 1: Fig. S3, the effect is similar to that with SUV_max_.

Similar studies were conducted by other researchers. Mattoli et al. [14]. compared data from Biograph mCT and Gemini GXL (Philips Medical System). Metabolic response (responder/non-responder) was evaluated twice (with and without harmonization) according to EORTC response categories. They found responders based on a harmonized classification with longer disease-free survival than responders based on a non-harmonized classification. Rubello et al. [25] validated EQ.PET for SUV harmonization with seven patients scanned on mCT and subsequently scanned on GE Discovery STE (General Electric, Milwaukee, WI). Their preliminary results demonstrated that EQ.PET was an easy and precise solution to harmonizing SUVs between different PET/CT scanners, decreasing SUV_max_ discrepancies from 149 to below 10%.

This study had several limitations. First, patients were generally scanned on same PET systems for therapy monitoring and follow-up, so patient number scanned on different PET systems is limited. And as a retrospective study, raw data available for second reconstruction is also limited. Hence, the patient population in all 3 groups was limited with cancer types varied, making it hard to evaluate therapy response using standard criteria. Second, our results verified that SUVs and TBRs could be harmonized across different PET systems and different reconstructions by applying EQ.PET, and the metabolic therapy responses were comparable after harmonization. But the accuracy of harmonized therapy response was not confirmed, since survival analysis was not performed in this study. Third, our results proved EQ.PET to be feasible in SUV harmonization regardless of lesion size and BMI of the patients. However, the nature of the retrospective study in one center with limited data makes the results less convincing. More extensive multicenter randomized controlled trials on single cancer type and thoroughly investigated cross-system comparisons may confirm our results and expand the opportunities for harmonized SUVs.

## Conclusions

Our study validated the use of EQ.PET for SUV harmonization in oncology patients across different reconstruction algorithms and different PET scanners. The results verified that EQ.PET is an efficient tool to harmonize SUVs and TBR, regardless of lesion size and BMI of the patients. With harmonized quantitative data, we may establish a unified standard for diagnosis, staging, follow-up, and therapy monitoring.

## Supplementary Information

**Additional file 1.** Supplementary Figures and Tables.

## Data Availability

The datasets used and/or analyzed during the current study are available from the corresponding author on reasonable request.

## Abbreviations

DFS: Disease-free survival
LoA: Limit of agreement
OSEM: Ordered subset expectation maximization
RC: Recovery coefficient
RMSE: Root mean square error
T/B: Tumor-to-blood
TBR: Tumor-to-background
TL: Tumor-to-liver
VOI: Volume of interest
