# Supplementary material for: Harmonization of standard uptake values across different positron emission tomography/computed tomography systems and different reconstruction algorithms: validation in oncology patients
Source: EJNMMI Phys. 2023 Mar 15;10:19. doi: 10.1186/s40658-023-00540-z (PMC10017904; doi:10.1186/s40658-023-00540-z)
Supplement: Supplementary file 1 — Additional file 1. Supplementary Figures and Tables. [file 40658_2023_540_MOESM1_ESM.docx]

**Supplementary Information**

**Harmonization of standard uptake values across different positron emission tomography/computed tomography systems and different reconstruction algorithms:
validation in oncology patients**

Yufei Song^1^, Xiangxi Meng^1^, Zhen Cao^2^, Wei Zhao^1^,
Yan Zhang^1^, Rui Guo^1^, Xin Zhou^1^, Zhi Yang^1,*^, Nan Li^1,*^

1. Key Laboratory of Carcinogenesis and Translational Research (Ministry of Education/Beijing), Key Laboratory for Research and Evaluation of Radiopharmaceuticals (National Medical Products Administration), Department of Nuclear Medicine, Peking University Cancer Hospital & Institute

2. Siemens Healthineers Ltd.

Corresponding authors:

Zhi Yang, pekyz@163.com

Nan Li, rainbow6283@sina.com


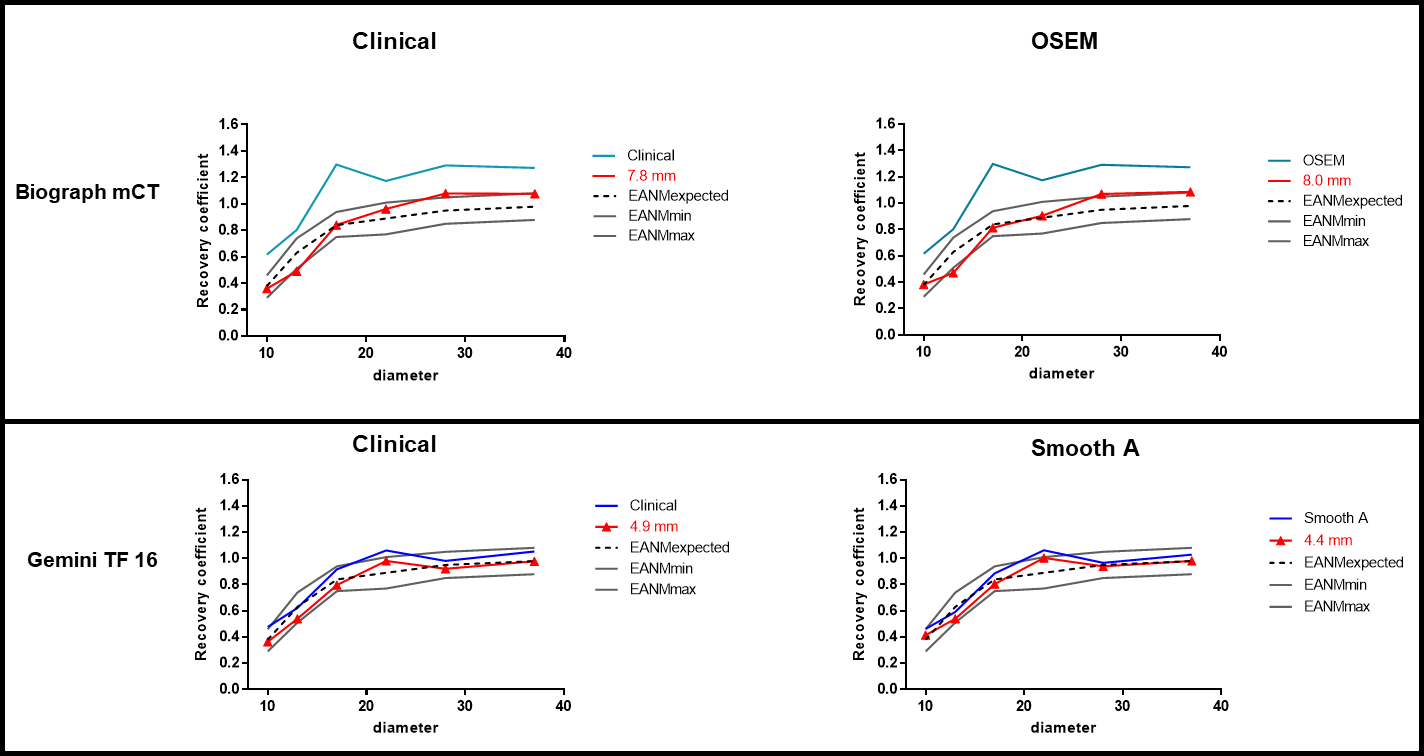


**Supplementary Fig. 1** Recovery coefficient curves obtained using NEMA PET Body Phantom acquired in Biograph mCT (upper panel) Gemini TF 16 (lower panel). (The red curve is the chosen EQ.PET filter for the specific reconstruction parameter)

**
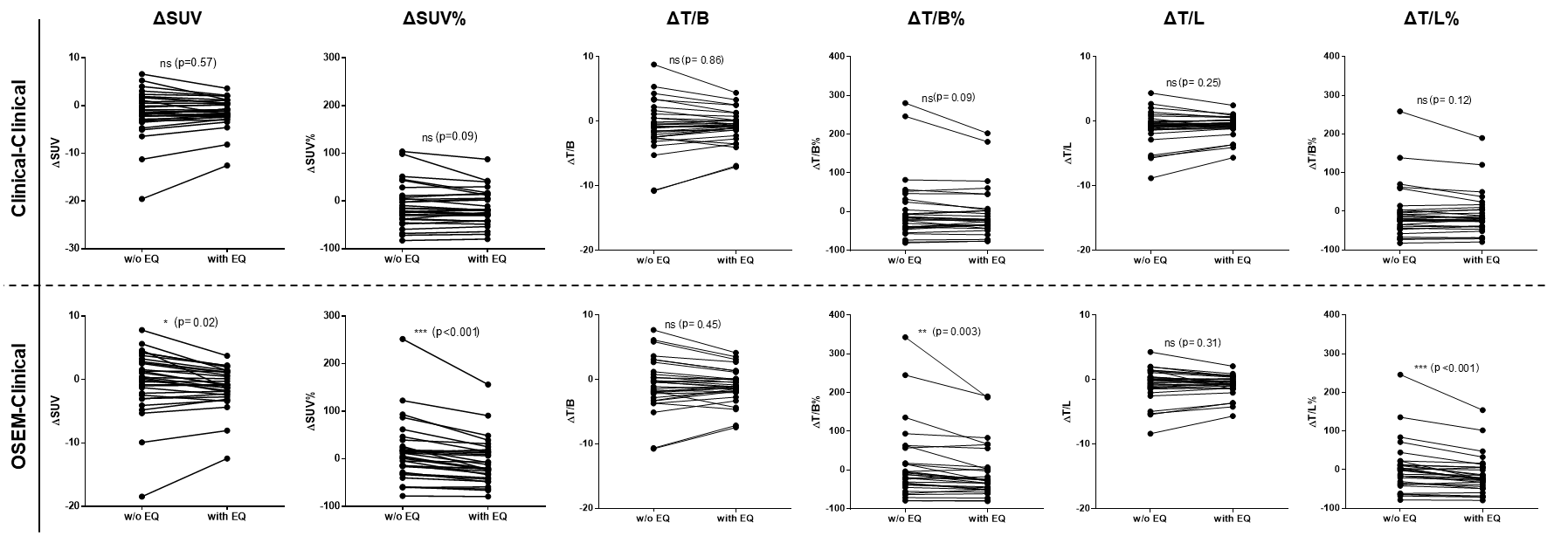
**

**Supplementary Fig. 2** Comparison of ΔSUV, ΔSUV%, and ΔTBR, ΔTBR% between data with and without harmonization in patients scanned twice on mCT in **Group 3**.


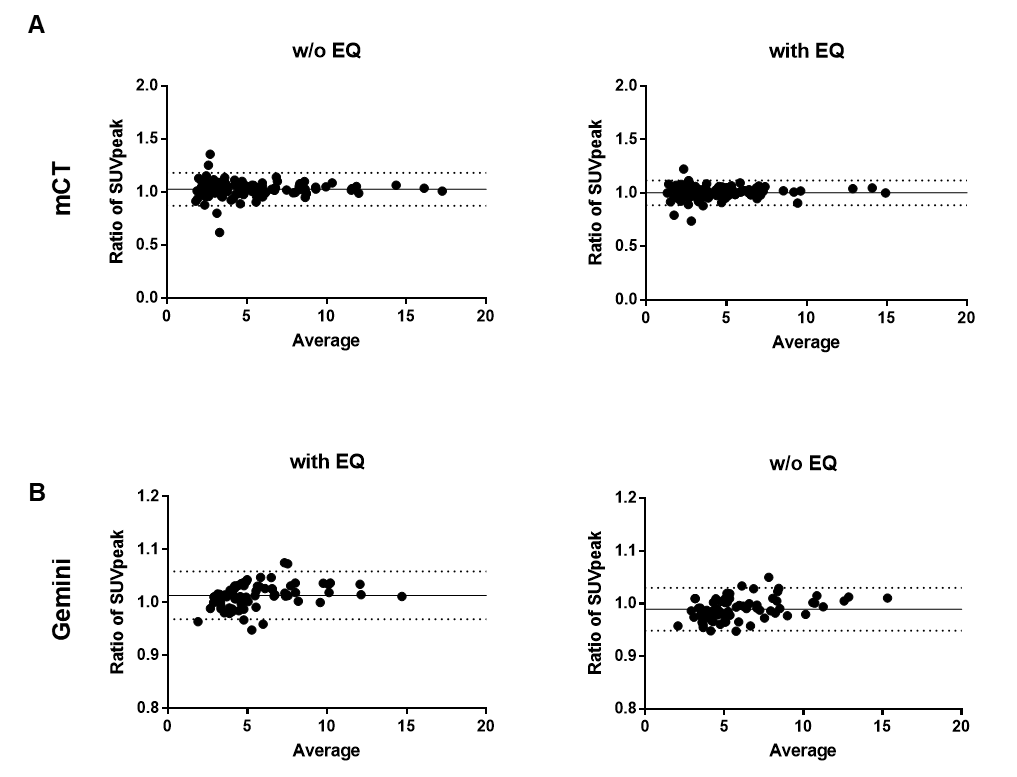


**Supplementary Fig. 3** Bland-Altman analysis of SUVpeak in lesions between data reconstructed using different reconstruction parameters acquired on mCT (A) and Gemini (B) in **Group 1.**

**Supplementary Table 1** Patient demography of **Group 1**

Patients scanned on 1 scanner with raw data reconstructed using 2 parameters

| **Patient No.** | **Gender** | **BMI** | **Age** | **Caner type** | **Lesions included** | **PET scanner** |
| --- | --- | --- | --- | --- | --- | --- |
| 1 | F | 22.9 | 68 | Gallbladder carcinoma | 18 | mCT |
| 2 | M | 23.8 | 61 | Hepatic cancer | 9 | mCT |
| 3 | M | 22.9 | 62 | Esophagus cancer | 4 | mCT |
| 4 | F | 20.4 | 55 | Hepatic cancer | 7 | mCT |
| 5 | F | 20.6 | 73 | Pancreatic cancer | 10 | mCT |
| 6 | M | 26.7 | 68 | Squamous cell lung carcinoma | 32 | mCT |
| 7 | F | 24.3 | 51 | Pulmonary adenocarcinoma | 1 | mCT |
| 8 | F | 24.3 | 63 | Non-Hodgkin lymphoma | 9 | mCT |
| 9 | M | 21.2 | 66 | Squamous cell lung carcinoma | 2 | mCT |
| 10 | M | 21.3 | 39 | Rectal adenocarcinoma | 3 | mCT |
| 11 | F | 26.4 | 61 | Malignant melanoma | 2 | mCT |
| 12 | F | 22.5 | 60 | Pulmonary adenocarcinoma | 1 | mCT |
| 13 | M | 27 | 71 | Pulmonary adenocarcinoma | 2 | mCT |
| 14 | M | 18.6 | 52 | Squamous cell lung carcinoma | 3 | mCT |
| 15 | F | 22.2 | 66 | Pulmonary adenocarcinoma | 3 | mCT |
| 16 | F | 23.7 | 46 | Pulmonary adenocarcinoma | 1 | mCT |
| 17 | M | 21.4 | 69 | Squamous cell lung carcinoma | 1 | mCT |
| 18 | F | 22.3 | 63 | Pulmonary adenocarcinoma | 2 | mCT |
| 19 | M | 24.9 | 64 | Squamous cell lung carcinoma | 1 | mCT |
| 20 | M | 23.4 | 55 | Pulmonary adenocarcinoma | 2 | mCT |
| 21 | M | 27.6 | 52 | Diffuse large B cell lymphoma, germinal center B-cell-like lymphoma | 5 | mcT |
| 22 | M | 21 | 50 | Pulmonary adenocarcinoma | 8 | mcT |
| 23 | M | 19.4 | 66 | Lung Cancer | 8 | mcT |
| 24 | M | 28.4 | 54 | Non-Hodgkin lymphoma | 13 | Gemini TF 16 |
| 25 | M | 29.1 | 57 | Esophagus cancer | 3 | Gemini TF 16 |
| 26 | M | 21.7 | 59 | Diffuse large B cell lymphoma, germinal center B-cell-like lymphoma | 6 | Gemini TF 16 |
| 27 | M | 20.5 | 69 | Non-Hodgkin lymphoma | 2 | Gemini TF 16 |
| 28 | M | 23.7 | 60 | Esophagus cancer | 5 | Gemini TF 16 |
| 29 | F | 21.3 | 70 | Non-Hodgkin lymphoma | 6 | Gemini TF 16 |
| 30 | M | 26 | 85 | Lung cancer | 2 | Gemini TF 16 |
| 31 | M | 24.2 | 64 | Esophagus cancer | 5 | Gemini TF 16 |
| 32 | F | 17.5 | 57 | non-Hodgkin lymphoma | 10 | Gemini TF 16 |
| 33 | F | 27.7 | 46 | Breast cancer | 4 | Gemini TF 16 |
| 34 | M | 21.5 | 57 | Colon cancer | 6 | Gemini TF 16 |
| 35 | M | 22.5 | 58 | Lung cancer | 7 | Gemini TF 16 |
| 36 | F | 29.2 | 60 | Lung cancer | 3 | Gemini TF 16 |
| 37 | F | 22.5 | 57 | Colon cancer | 5 | Gemini TF 16 |
| 38 | M | 23.5 | 52 | Lung cancer | 2 | Gemini TF 16 |

**Supplementary Table 2** Patient demography of **Group 2**

Patients received PET scans on different scanner for follow-up or therapy monitoring

| **Patient No.** | **Gender** | **Age** | **Cancer type** | **Treatment type** | **1^st^ scanner** | **2^nd^ scanner** | **Interval between PET scans (days)** | **Lesions included** |
| --- | --- | --- | --- | --- | --- | --- | --- | --- |
| 1 | F | 23 | Hodgkin lymphoma | unknown | Gemini TF | mCT | 56 | 1 |
| 2 | F | 55 | non-Hodgkin lymphoma | Chemotherapy+targeted therapy | Gemini TF | mCT | 42 | 1 |
| 3 | F | 44 | non-Hodgkin lymphoma | Chemotherapy | Gemini TF | mCT | 42 | 1 |
| 4 | F | 62 | non-Hodgkin lymphoma | Chemotherapy | Gemini TF | mCT | 43 | 3 |
| 5 | M | 63 | Squamous cell lung carcinoma | Chemotherapy | Gemini TF | mCT | 104 | 1 |
| 6 | M | 72 | Esophagus squamous carcinoma | chemotherapy+radiotherapy | mCT | Gemini TF | 38 | 2 |
| 7 | F | 67 | non-Hodgkin lymphoma | chemotherapy | mCT | Gemini TF | 149 | 3 |
| 8 | F | 62 | non-Hodgkin lymphoma | no treatment | mCT | Gemini TF | 50 | 6 |
| 9 | F | 44 | Diffuse large B cell lymphoma | unknown | mCT | Gemini TF | 41 | 3 |
| 10 | F | 40 | Hodgkin lymphoma | chemotherapy | mCT | Gemini TF | 139 | 1 |
| 11 | M | 41 | non-Hodgkin lymphoma | chemotherapy | mCT | Gemini TF | 54 | 5 |

**Supplementary Table 3** Patient demography of **Group 3**

Patients received 2 PET scans on mCT for follow-up or therapy monitoring

| **Patients No.** | **Gender** | **Age** | **Cancer type** | **Treatment type** | **Interval of 2 PET scans (days)** | **Lesions included** |
| --- | --- | --- | --- | --- | --- | --- |
| 1 | F | 51 | Pulmonary adenocarcinoma | chemotherapy | 126 | 1 |
| 2 | F | 63 | non-Hodgkin lymphoma | chemotherapy | 91 | 9 |
| 3 | M | 66 | Squamous cell lung carcinoma | chemotherapy | 63 | 2 |
| 4 | M | 39 | Rectal adenocarcinoma | chemotherapy+radiotherapy | 195 | 3 |
| 5 | F | 61 | Malignant melanoma | chemotherapy | 82 | 2 |
| 6 | F | 60 | Pulmonary adenocarcinoma | chemotherapy | 237 | 1 |
| 7 | M | 71 | Pulmonary adenocarcinoma | targeted therapy (Gefitinib) | 96 | 2 |
| 8 | M | 52 | Squamous cell lung carcinoma | chemotherapy | 87 | 3 |
| 9 | F | 66 | Pulmonary adenocarcinoma | no treatment | 104 | 3 |
| 10 | F | 46 | Pulmonary adenocarcinoma | chemotherapy | 39 | 1 |
| 11 | M | 69 | Squamous cell lung carcinoma | chemotherapy+targeted therapy | 18 | 1 |
| 12 | F | 63 | Pulmonary adenocarcinoma | targeted therapy (Gefitinib) | 189 | 2 |
| 13 | M | 64 | Squamous cell lung carcinoma | chemotherapy +immunotherapy (PD-L1) | 92 | 1 |
| 14 | M | 55 | Pulmonary adenocarcinoma | chemotherapy+targeted therapy | 114 | 2 |

**Supplementary Table 4** Measured values in image pairs of **Group 3**

| **Mean Ratio**  **(95% LoA*)** |  | **ΔSUV** | **ΔT/B** | **ΔT/L** |
| --- | --- | --- | --- | --- |
|  | w/o EQ | 0.07 | 1.11 | 1.23 |
|  |  | (-8.70~8.84) | (-1.18~3.40) | (-7.92~10.39) |
|  | with EQ | 1.02 | 0.88 | 1.14 |
|  |  | (0.23~1.81) | (-0.55~2.31) | (-0.43~2.70) |
| **Mean difference**  **(95% LoA*)** |  | **ΔSUV%** | **ΔT/B%** | **ΔT/L%** |
|  | w/o EQ | -14.38 | -8.30 | -6.85 |
|  |  | (-36.10~7.34) | (-60.37~43.76) | (-33.09~19.40) |
|  | with EQ | -1.41 | 3.32 | 4.66 |
|  |  | (-11.27~8.46) | (-13.89~20.53) | (-11.19~20.52) |

* LoA: limits of agreement
